# The mechanism and candidate compounds of aged citrus peel (*chenpi*) preventing chronic obstructive pulmonary disease and its progression to lung cancer

**DOI:** 10.29219/fnr.v65.7526

**Published:** 2021-05-17

**Authors:** Lin Zhou, Wenwen Gu, Fuguang Kui, Fan Gao, Yuji Niu, Wenwen Li, Yaru Zhang, Lijuan Guo, Junru Wang, Zhenzhen Guo, Gangjun Du

**Affiliations:** 1 Institute of Pharmacy, Pharmaceutical College of Henan University, Kaifeng, China; 2 School of Pharmacy and Chemical Engineering, Zhengzhou University of Industry Technology, Xinzheng, China

**Keywords:** chenpi, chronic obstructive pulmonary disease (COPD), lung cancer, hesperetin, network pharmacology

## Abstract

**Background:**

Chronic obstructive pulmonary disease (COPD) is an important risk factor for developing lung cancer. Aged citrus peel (*chenpi*) has been used as a dietary supplement for respiratory diseases in China.

**Objective:**

To explore the mechanism and candidate compounds of *chenpi* preventing COPD and its progression to lung cancer.

**Methods:**

The active components and potential targets of *chenpi* were retrieved from the Traditional Chinese Medicine Systems Pharmacology (TCMSP) database. Disease-associated targets of COPD and lung cancer were collected in the Gene Cards and TTD database. The component-target network and PPI network were constructed using the Cytoscape 3.8.0 software. David database was used for GO and KEGG enrichment analysis. The main active components were verified by using the autodock Vina 1.1.2 software. Mouse lung cancer with COPD was induced by cigarette smoking (CS) combined with urethane injection to confirm preventing the effect of hesperetin (the candidate compound of *chenpi*) on COPD progression to lung cancer and its underlying mechanisms.

**Results:**

The network analysis revealed that the key active components of *chenpi* (nobiletin, naringenin, hesperetin) regulate five core targets (AKT1, TP53, IL6, VEGFA, MMP9). In addition, 103 potential pathways of *chenpi* were identified. *Chenpi* can prevent COPD and its progression to lung cancer by getting involved in the PI3K-Akt signaling pathway and MAPK signaling pathway. Molecular docking indicated that hesperetin had better binding activity for core targets. In mouse lung cancer with COPD, treatment with hesperetin dose-dependently improved not only lung tissue injury in COPD but also carcinoma lesions in lung cancer. Meanwhile, hesperetin could suppress the protein expression of AKT1, IL6, VEGFA, MMP9 and up-regulate the protein expression of TP53, and thus reduced the risk of COPD progression to lung cancer.

**Conclusion:**

Hesperetin is a candidate compound of *chenpi* that helps in preventing COPD and its progression to lung cancer by regulating AKT1, IL6, VEGFA, MMP9 and TP53.

## Popular scientific summary

- Chenpi can prevent COPD and its progression to lung cancer by getting involved in the PI3K-Akt signaling pathway and MAPK signaling pathway.
- Hesperetin is a candidate compound of *chenpi* that helps in preventing COPD and its progression to lung cancer.
- Hesperetin could suppress the protein expression of AKT1, IL6, VEGFA, MMP9 and up-regulate the protein expression of TP53 to reduce the risk of COPD progressing to lung cancer.

Chronic obstructive pulmonary disease (COPD) is a common disease characterized by persistent airflow limitation and chronic inflammation of the airways (1). Lung cancer is the most frequent cause of cancer-related deaths worldwide and is also one of the most malignant tumors reported in recent 5 years (2). In clinical practice, it has been found that lung cancer usually coexists with COPD (3), and COPD may be a potential risk factor for the development of lung cancer (4). In one screening study, the vast majority of lung cancers (94%) appeared in patients with early-stage COPD (5). Powell et al. conducted a retrospective analysis of the lung cancer risk of COPD patients and found that 23% of the 11,888 lung cancer cases were diagnosed with COPD (6). COPD has the features of chronic lung injury; there are two main performance related issues: limited airflow and lung parenchyma destruction, which are often accompanied by an increase in cell apoptosis, autophagy and aging (7). By contrast, lung cancer is characterized by abnormal DNA damage and repair, which is often accompanied by angiogenesis, genomic instability, cell proliferation and weakened immunity (8). However, cigarette smoking is the highest risk factor for the two diseases (9). Many studies have shown that immune dysfunction, dysregulation of lung microbiota, inflammatory infection, oxidative stress and DNA damage play a significant role in the development of COPD and lung cancer, and these reasons may be the potential driving factors of COPD progression to lung cancer (10). Clinically, current treatment drugs for COPD include bronchodilators, broad-spectrum anti-inflammatory agents and glucocorticoids, etc. However, these treatments exhibit many limitations because of the resistance and side effects. Therefore, the development of novel and efficient drugs is urgently required.

Aged citrus peel (*chenpi*) is the dry peel of the plant *Citrus reticulata*, Blanco obtained after an aging processing (11, 12). *Chenpi* is not only consumed as a dietary supplement, but also used for the treatment of respiratory disease by regulating qi, strengthening the spleen, drying dampness, resolving phlegm and reliving cough (13). Modern pharmacological studies have shown that *chenpi* has anti-inflammatory, anti-cancer, anti-oxidative, sputum diluting and other effects (14). In recent years, the research related to effect of *chenpi* on cancer is increasing, especially for lung cancer (15). Meanwhile, *chenpi* has an extraordinary role as a prescription for COPD treatment because of its dampness drying and phlegm resolving effects(16). Moreover, *chenpi* did not show drug resistance and side effects compared with bronchodilators and glucocorticoids for COPD. To date, more than 140 compounds have been isolated and identified from *chenpi*; these predominantly include flavonoids, volatile oils, alkaloids and other trace elements (17). Nevertheless, the mechanism and main compounds of *chenpi* preventing COPD progressing to lung cancer are not yet clear enough.

**Fig. 1:**
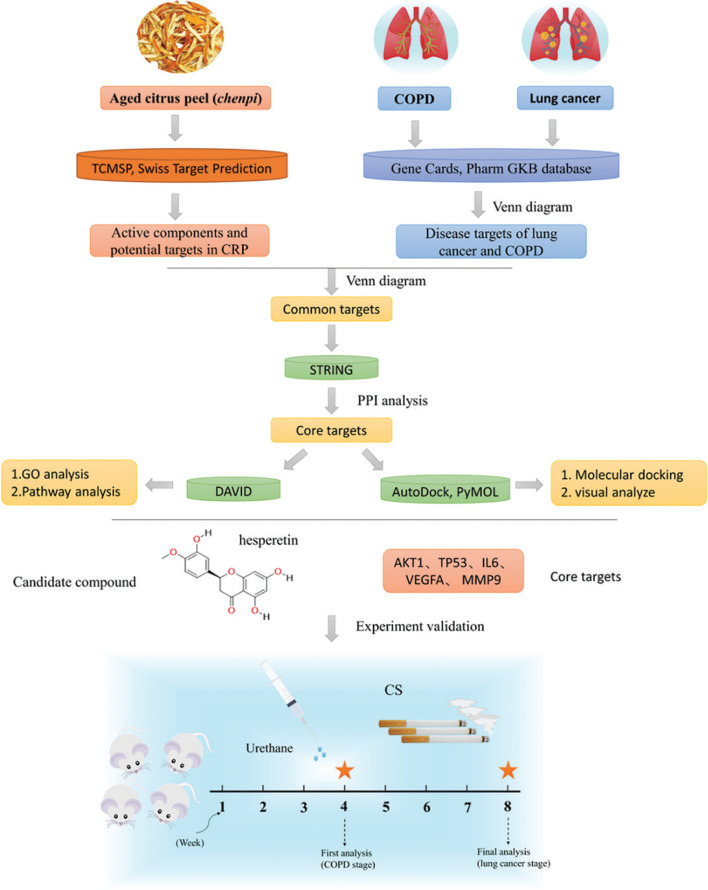
The research scheme in this experiment.

*Chenpi* has the characteristics of multiple components and multiple targets, and analyzing this complexity can be achieved through network pharmacology analysis (18, 19). This study will make use of network pharmacology and mouse lung cancer with COPD to elucidate the mechanism and candidate compounds of *chenpi* as a potential dietary supplementation for prevention of COPD and its progression to lung cancer (Fig. 1).

## Materials and methods

### Active components and potential targets in chenpi

We collected the active components and potential targets of *chenpi* from TCMSP (http://tcmspw.com), which is a system pharmacology platform designed for comprehensively studying TCMs. With ‘aged citrus peel/*chenpi*/tangerine peel’ as the key words, the screening conditions were as follows: oral bioavailability (OB) ≥ 30% and drug-likeness (DL) ≥ 0.18, which are the most important indicators for evaluating the characteristics of absorption, distribution, metabolism and excretion (ADME) (20). The potential targets of *chenpi* are also obtained from the Swiss Target Prediction (http://www.swisstargetprediction.ch/) database as the supplement and then corrected through the UniProt database (https://www.uniprot.org); these are limited to species of *Homo sapiens*.

### Disease-associated targets of lung cancer and COPD

Disease-associated targets of lung cancer and COPD were obtained through the Gene Cards database (https://www.genecards.org) and the Pharm GKB database (https://geneticassociationdb.nih.gov/), making use of the keywords ‘lung cancer’ and ‘COPD’. A Venn diagram was used to demonstrate the intersections of targets between COPD and lung cancer.

### Construction of the components-targets network

To acquire candidate targets of *chenpi* preventing COPD and its progression to lung cancer, we integrated the *chenpi*-related targets with target genes of lung cancer and COPD and chose those replicate genes as candidate targets. Then the components–targets (candidate targets) network of *chenpi* was constructed through the network visualization Cytoscape3.7.0 software.

### Construction of the PPI network

The targets of *chenpi* preventing COPD progression to lung cancer were imported into the string biological database (https://string-db.org/), the species was limited to ‘Homo sapiens’ and the minimum confidence score > 0.7. The discrete network nodes were hidden to obtain the protein interaction data information of intersection targets. The Cytoscape3.7.0 software was used to build the PPI network and screen key targets. The three topological features (‘Degree’, ‘Betweenness centrality’ and ‘Closeness centrality’) were used to calculate the importance of each target (21, 22).

### Gene ontology and pathway enrichment analysis

To further explore the multiple mechanisms of *chenpi* preventing COPD and its progression to lung cancer from the system level, GO enrichment analysis including Biological Process, Cellular Component, Molecular Function and KEGG pathway enrichment analysis were performed using the DAVID (https://david.ncifcrf.gov/)database. The results of top 10 GO enrichment analysis and top 15 KEGG pathway enrichment analysis were selected (*P* < 0.05).

### Molecular docking of active components and targets of chenpi

The Mol2 molecular structure of chemical constituents from *chenpi* was obtained from the TCMSP database. The three-dimensional (3D) structure of key target genes was downloaded from the RCSB PDB database (https://www.rcsb.org/). The molecular structure documents were converted into PDBQT format by using the autodock tools 1.5.6 software, and molecular docking was performed using autodock Vina 1.1.2 software. The PyMOL 2.3.2 software was used to visually analyze the results with higher docking scores.

## Materials

Hesperetin (CAS# 520-33-2, purity> 98% via HPLC) was purchased from Shanghai Yuanye Biological Technology (Shanghai, China). Urethane was purchased from Sigma Chemical Co (St Louis, MO, USA). Antibodies including anti-AKT1, anti-IL6, anti-VEGF, anti-EGFR, anti-TP53, anti-GAPDH and fluorescein isothiocyanate (FITC)-conjugated antimouse immunoglobulin G (IgG) were obtained from Proteintech Group, Inc (Wuhan, China). HRP-conjugated goat anti-mouse IgG polyclonal antibody was obtained from R&D Systems.

### Animals

Seven-week-old female ICR mice with body weight of 23±2 g (n=90) were obtained from the Henan Provincial Medical Laboratory Animal Center. All mice were housed in individual ventilated cages. Animals were fed standard rodent chow and water. Standard rodent chow was purchased from Henan the Provincial Medical Laboratory Animal Center (Zhengzhou, China), License No. SCXK (YU) 2015-0005, Certificate No. 41000100002406. All animal procedures were approved by the Animal Experimentation Ethics Committee of Henan University (permission number HUSAM 2016-288), and in accordance with the Helsinki Declaration of 1975, as revised in 2008.

### Cigarette smoking and urethane induced lung cancer with COPD

Mice received an intraperitoneal injection of urethane (dissolved in sterile 0.9% NaCl, 600 mg/kg body weight) once weekly for 8 weeks. The next day after the urethane injection, the mice were exposed to cigarette smoking (1 cigarette at a time for 1 h, twice a day and 6 days per week) in a whole-body exposure system (30 cm × 40 cm × 60 cm’s chamber) for 8 weeks and the normal mice were exposed to normal air in a respective exposure system. Following the first urethane injection, mice received hesperetin (25, 50, 100 mg/kg/day) via intragastric administration once per day for 8 weeks. The health of the mice was monitored daily, and body weights were measured weekly. Lung function was analyzed weekly by tidal volume (TV) using the animal respiratory metabolic measurement system (Sable Systems International, United States). At 4 weeks (COPD stage) and 8 weeks (lung cancer stage) after the first urethane injection, mice were sacrificed under anaesthesia with pentobarbital sodium (90 mg/kg). Average lung carcinomas per mouse were counted in the lung cancer stage. The lung tissue injury was analyzed by histopathological evaluation of H&E staining and lung injury score. Pulmonary edema formation was measured via determination of the lung W/D ratio. Western blot and immunofluorescence analyses were used to confirm underlying mechanisms of hesperetin preventing COPD and its progression to lung cancer.

### Histopathological evaluation

A part of each lung was preserved in 10% buffered formalin and routinely embedded in paraffin. Lung sections (5 μm) were stained with haematoxylin and eosin (H&E). Lung pathology scored the lung injury according to previously published criteria (23). The mean score from five examined fields was calculated as the injury score.

### Lung W/D ratio

The mice in COPD stage were killed humanely, lung tissues were collected and weighed immediately (the wet weight) and were then heated at 80°C for 48 h to obtain the dry weight. The lung wet/dry (W/D) ratio was calculated.

### Western blot analysis

Lung extracts were prepared in a RIPA cell lysis buffer, the equal proteins were separated using 12% sodium dodecyl sulphate–polyacrylamide gel electrophoresis and then transferred to polyvinylidene fluoride membranes (Millipore, Germany) and probed with antibodies against AKT1, IL6, VEGFA, MMP9, TP53 and GAPDH. Antibody binding was detected via enhanced chemiluminescence according to the manufacturer’s instructions (Pierce, Rockford, IL). Band density was quantified using ImageJ software (NIH, Bethesda, MD, USA) and normalized to the corresponding control group.

### Immunofluorescence staining

Lung tissues sections were treated with blocked with 5% BSA for 30 min at room temperature and incubated with anti-AKT1, anti-IL6, anti-VEGFA, anti-MMP9, anti-TP53 and secondary antibody (FITC-labelled goat anti-rabbit IgG). Then the sections were fixed with anti-fluorescence quencher and photographed under the fluorescence microscope. Fluorescence intensity was quantified using Image J software (NIH, Bethesda, MD, USA).

### Statistical analyses

Data were presented as the mean ± SD and statistically analyzed using GraphPad Prism, Version 7.0 (San Diego, CA, USA). Difference between two groups was evaluated using *t* test. A *P* value of less than 0.05 was considered statistically significant.

## Results

### Screening of active components and potential targets in chenpi

**Fig. 2:**
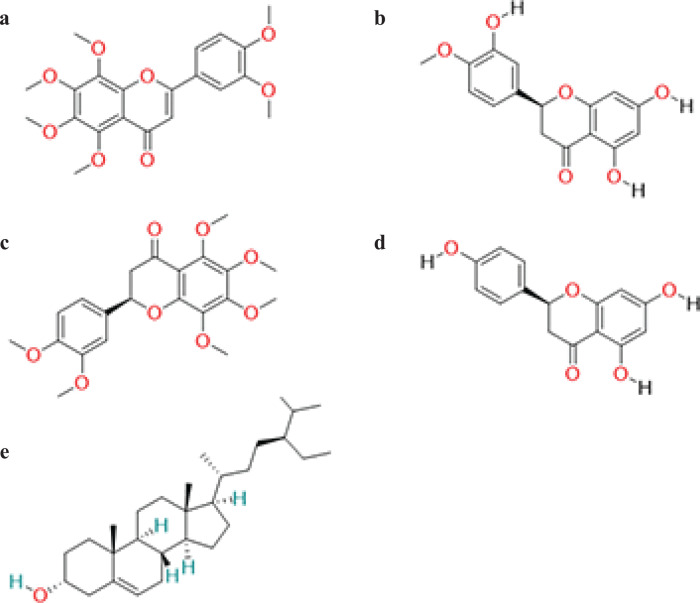
The 2D structure of active components in *chenpi.* (a) The 2D structure of nobiletin. (b)The 2D structure of hesperetin. (c) The 2D structure of citromitin. (d) The 2D structure of naringenin. (e) The 2D structure of sitosterol.

A total of 63 ingredients were obtained and five active compounds were ultimately chosen for further investigation using OB ≥30% and DL ≥0.18 as screening conditions. The five components are nobiletin (Fig. 2a) hesperetin (Fig. 2b), citromitin (Fig. 2c), naringenin (Fig. 2d) and sitosterol (Fig. 2e). A total of 251 targets were identified for five compounds, deleting duplicate targets of the same compound.

### Screening of disease-associated targets of lung cancer and COPD

**Fig. 3:**
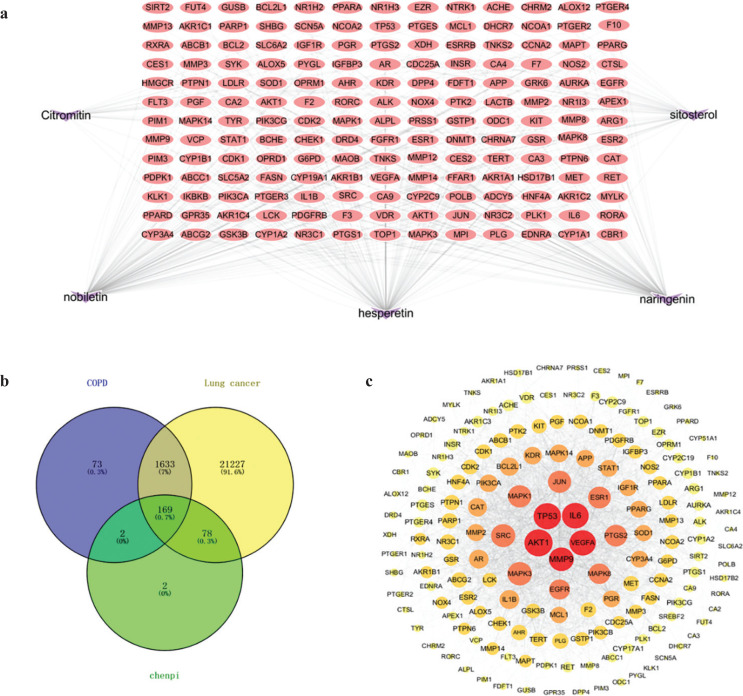
The network of *chenpi*-COPD and lung cancer intersection targets. (a) The components–targets network. A network with 174 nodes and 342 edges linking five compounds in *chenpi* and 169 target genes. The red nodes represent the targets, the purple nodes represent the compounds. (b) Venn diagram. (c) The PPI network containing 169 nodes and 1,992 edges. The node represents the protein, and the edge represents the interaction between proteins.

In the Gene Cards database and Pharm GKB database, 1,877 of COPD disease targets and 23,107 of lung cancer disease targets were obtained. There were 1,802 common targets in COPD and lung cancer. (Fig. 3b).

### Components–targets networks analysis

We identified 169 common targets of *chenpi* preventing COPD and its progression to lung cancer (Fig. 3b). For displaying intuitive interactions in *chenpi*, the components–targets network contained the five ingredients and 169 targets, including 174 nodes and 342 edges (Fig. 3a). The active components of *chenpi* with the most target genes are nobiletin, naringenin and hesperetin (Table 1), which may be the key components of *chenpi* preventing COPD and its progression to lung cancer.

**Table 1:** Information of five active ingredients of chenpi and number of corresponding targets

MOL ID	Molecule name	OB%	DL	Target number
MOL005815	Citromitin	86.90	0.51	27
MOL005828	Nobiletin	61.67	0.52	124
MOL004328	Naringenin	59.29	0.21	122
MOL000359	Sitosterol	36.91	0.75	52
MOL002341	hesperetin	70.31	0.27	126

### PPI network analysis

In the Cytoscape 3.8.0 software, the PPI network of the 169 targets was established (Fig. 3c). This PPI network contains 169 nodes and 1,992 edges, and the average degree of nodes is 23.7. The node represents the protein, and the edge represents the interaction between proteins; the degree determines the node area, and the larger the node area, the greater the role of the protein in the network; the color of the node represents the interaction degree of the node, and the lighter the color of the node from the inside to the outside, the lower the interaction between the proteins. Five genes were identified with higher values of ‘Degree’ (above twofold of the median value), ‘Betweenness centrality’ and ‘Closeness centrality’ (above the median value) as the key targets of *chenpi* preventing COPD and its progression to lung cancer, they were AKT1, IL6, VEGFA, MMP9 and TP53 (Table 2).

**Table 2:** The targets of *chenpi* preventing chronic obstructive pulmonary disease and its progression to lung cancer and its relevant topological parameters

Uniprot ID	Target	Betweenness centrality	Closeness centrality	Degree
P31749	AKT1	0.07	0.71	99
P04637	TP53	0.07	0.69	94
P05231	IL6	0.08	0.68	91
P15692	VEGFA	0.06	0.68	90
P14780	MMP9	0.02	0.60	60

### GO biological process and KEGG pathway enrichment analysis

**Fig. 4:**
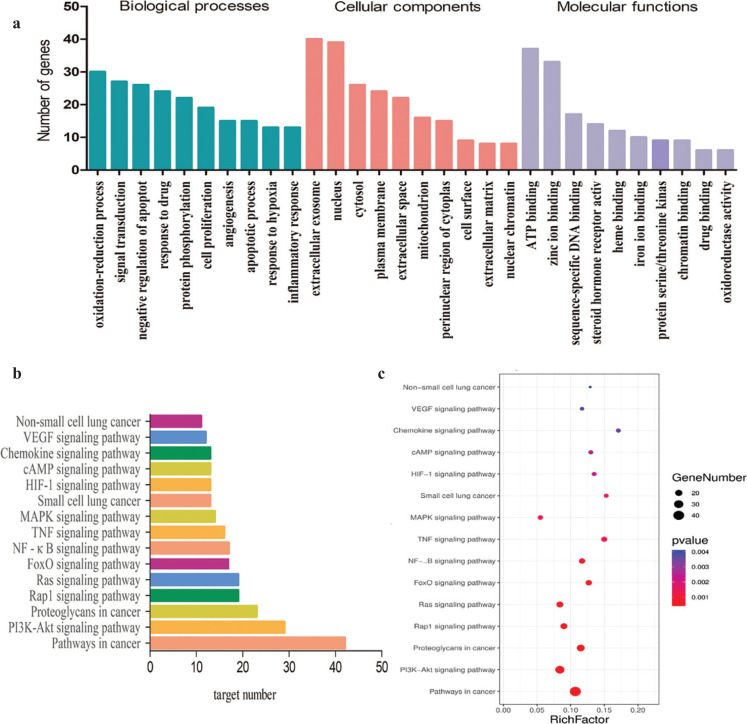
GO and KEGG pathway enrichment analysis. (a) Histogram of GO enrichment analysis. (b) Histogram of KEGG enrichment analysis. (c)Visualization of KEGG enrichment analysis.

In GO and KEGG enrichment analysis, the screened standard of *P* value <0.05, we obtained 419 GO enrichment items, including 360 biological processes, mainly involving the oxidation reduction process, signal transfer, negative regulation of apoptotic process and positive regulation of cell promotion, protein phosphorylation; 37 molecular functions, mainly involving ATP binding, zinc ion binding, sequence specific DNA binding, steroid hormone receptor activity, heme binding and 22 cell compositions, mainly related to extractive exosomes, nucleus, plasma membrane, extracellular space, mitochondrion, etc. In the KEGG enrichment analysis, 103 related signaling pathways were obtained, mainly involving the PI3K-Akt signaling pathway, mitogen-activated protein kinase (MAPK) signaling pathway, etc. The histogram of the top 10 GO enrichment analysis (Fig. 4a) and the top 15 KEGG pathway enrichment analysis (Fig. 4b) were shown. The top 15 of KEGG enrichment analysis was visualized using Omicshare (http://www.omicshare.com/)(Fig. 4c).

### Molecular docking of active components and targets of chenpi

**Fig. 5:**
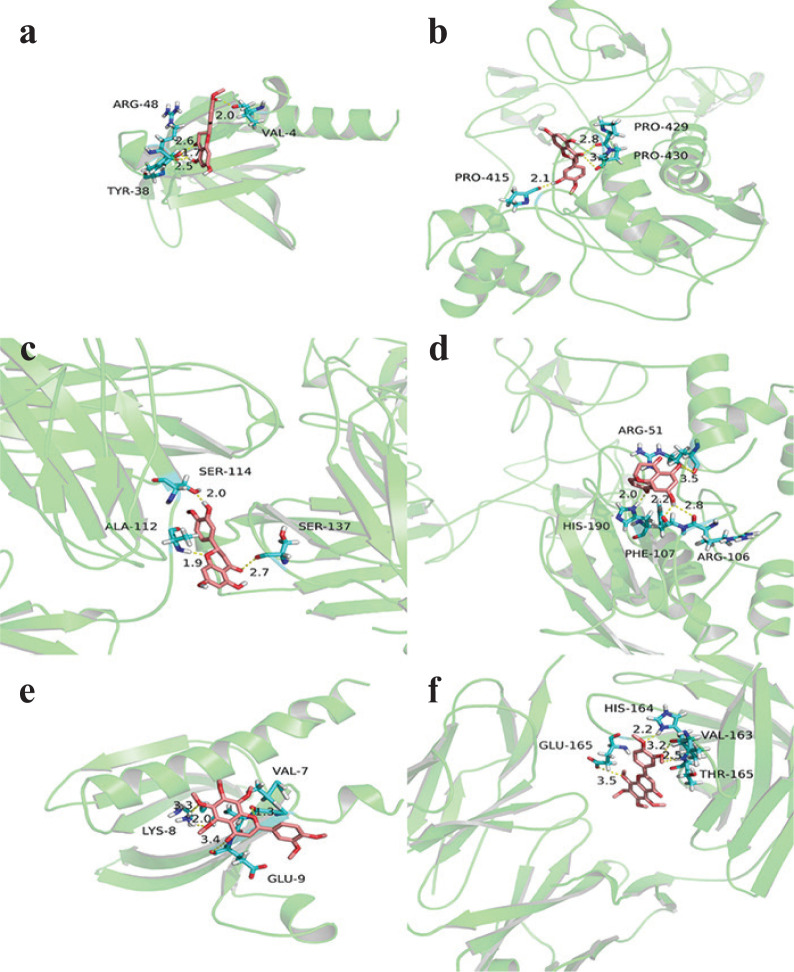
Pattern diagram of molecular docking. (a) Hesperetin-AKT1, (b) hesperetin-MMP9, (c) hesperetin-IL6, (d) naringenin-MMP9, (e) nobiletin-AKT1, (f) nobiletin-IL6.

The Auto Dock Vina 1.1.2 software was used to dock three active components of hesperetin, naringenin and nobiletin with five core targets respectively (Table 3). Binding energy less than 0 indicates spontaneous binding of the ligand and receptor, and the more stable the binding conformation, the lower the binding energy and the greater the possibility of action. It is generally believed that the binding energy greater than −5.0 kcal/mol indicates a good binding activity between the small molecule ligand and the receptor protein (24). The better docking results were selected for molecular docking visualization analysis with Pymol 2.3.2 software (Fig. 5). The dotted line in the figure is the hydrogen bond, and the value is the bond length. The results showed that hesperetin demonstrated the best activity. Hesperetin binds AKT1 to form hydrogen bonds with the three amino acids near the active site:ARG-48, VAL-4, TYR-38 (Fig. 5a); binds MMP9 to forms hydrogen bonds with the three amino acids near the active sites: PRO-429, PRO-430, PRO-415 (Fig. 5b); binds IL6 to form hydrogen bonds with the three amino acids near the active site: HER-114, ALA-104, SER-137 (Fig. 5c). Naringenin binds to MMP9 and interacts with the four amino acids ARG-51, ARG-106, HIS-190, PHE-107 near the active site to form hydrogen bonds (Fig. 5d). Nobiletin binds AKT1 to forms hydrogen bonds with the three amino acids GLU-9, HIS-164, LYS-8 near the active site (Fig. 5e); binds IL6 to form hydrogen bonds with the four amino acids of GLU-165, HIS-164, VAL-163, THR-165 near the active site (Fig. 5f).

**Table 3:** Molecular docking of main active ingredients of *chenpi* and core targets.

Ingredient	Target name	Protein Data Bank (PDB) ID	Energy (kcal/mol)
hesperetin	IL6	4cni	–7.2
hesperetin	MMP9	1l6j	–6.54
hesperetin	AKT1	1unq	–6.88
hesperetin	TP53	1gzh	–5.89
hesperetin	VEGFA	3v2a	–5.98
naringenin	MMP9	1l6j	–5.96
naringenin	AKT1	1unq	–4.83
naringenin	TP53	1gzh	–5.48
naringenin	VEGFA	3v2a	–5.4
naringenin	IL6	4cni	–5.48
nobiletin	MMP9	1l6j	–5.49
nobiletin	AKT1	1unq	–5.89
nobiletin	TP53	1gzh	–5.25
nobiletin	IL6	4cni	–5.96
nobiletin	VEGFA	3v2a	–4.93

### Hesperetin prevents COPD and its progression to lung cancer

**Fig. 6:**
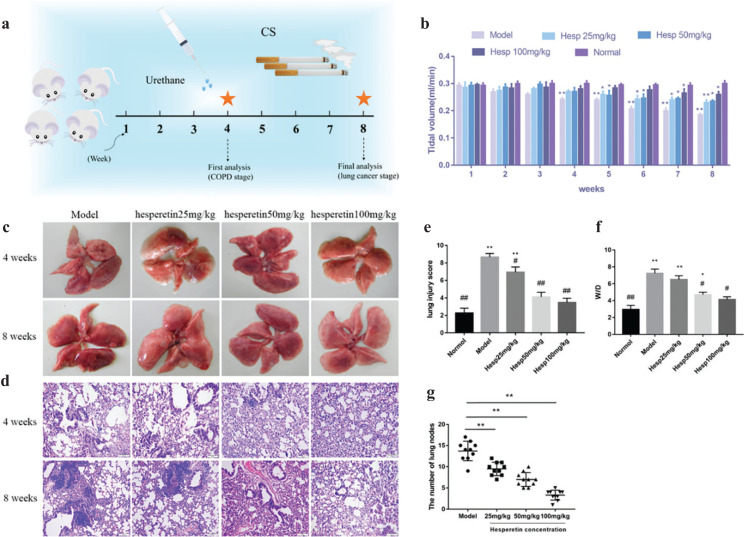
The preventive efficacy of hesperetin on urethane and CS-induced lung cancer with COPD. (a) Schematic design of the experimental procedure. (b) Tidal volume (TV) in lung function. (c) The whole lung at 4 and 8 weeks after urethane injection and CS exposure to the naked eye. (d) HE staining of lung tissues. (e) Lung injury score. (f) Lung W/D ratio. (g) The number of lung nodes at 8 weeks. The data are presented as the mean ± SD (n = 10), the experiments were repeated three times and statistical significance was determined by a *t* test. *P < 0.05, **P < 0.01 vs. normal; ^#^*P* < 0.05, ^##^*P* < 0.01 vs. the model.

To study the underlying pathogenic mechanisms of *chenpi* preventing COPD and its progression to lung cancer, urethane and CS-induced lung cancer with COPD was utilized (Fig. 6a). At 4 weeks, the TV in model mice decreased more than 20% (*P* < 0.01) (Fig. 6b), indicating development of COPD. At 8 weeks, lung cancer nodes were visible to the naked eye in the model group (Fig. 6c), suggesting a progression of COPD into lung cancer. H&E staining showed that the lung pathological changes were mainly manifested as enlarged alveolar space, thinner alveolar septum and destroyed alveolar wall in the model group at 4 weeks and at 8 weeks, cell structure was destroyed and nuclei were seriously condensed in the model group (Fig. 6c–d). However, compared to the model group, hesperetin treatment (25, 50, 100 mg/kg/day) dose-dependently increased the TV (*P* < 0.05) (Fig. 6b) and alleviated CS and urethane-induced lung pathologic changes at 4 and 8 weeks (Fig. 6c–d). Moreover, the lung injury and wet-to-dry weight ratio in the hesperetin group at 4 weeks was significantly reduced (*P* < 0.01) compared to the model group, indicating an attenuation of lung injury and lung edema (Fig. 6e–f). At 8 weeks, hesperetin treatment improved not only lung tissue injury but also carcinoma lesions (*P* < 0.01) (Fig. 6g). These results suggested that hesperetin as the candidate compound of *chenpi* could prevent COPD and its progression to lung cancer.

### Hesperetin could regulate the COPD and lung cancer related targets in urethane and CS induced lung cancer with COPD

**Fig. 7:**
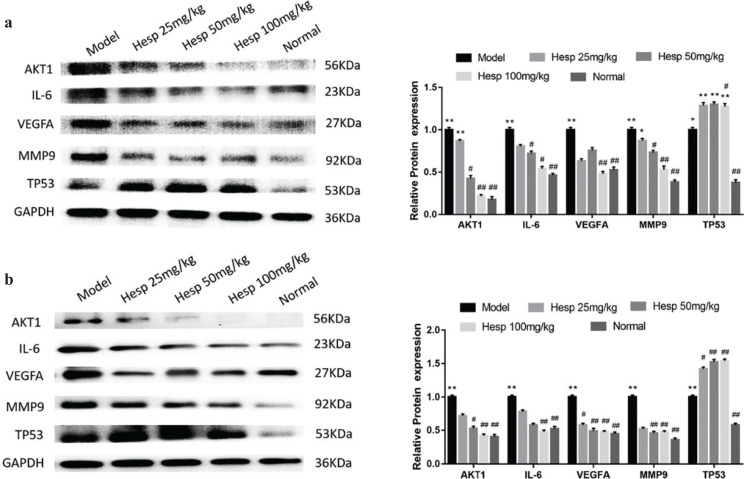
Hesperetin regulates the core targets of COPD and lung cancer in urethane and CS-induced lung cancer with COPD. (a) Protein expression of core targets examined by western blot in COPD stage. (b) Protein expression of core targets examined by western blot in the lung cancer stage. The data are presented as the mean ± SD (*n* = 10), the experiments were repeated three times and statistical significance was determined by a *t* test. *P < 0.05, **P < 0.01 vs. normal; ^#^*P* < 0.05, ^##^*P* < 0.01 vs. the model.

**Fig. 8:**
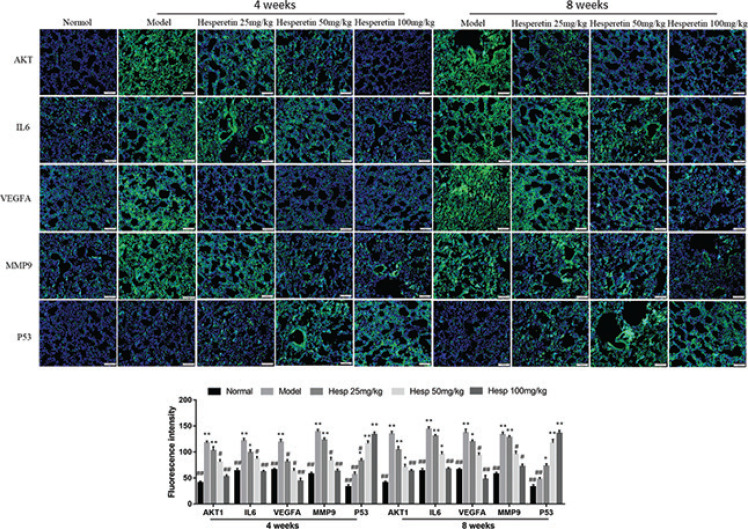
Hesperetin regulates the core targets of COPD and lung cancer in urethane and CS-induced lung cancer with COPD examined by immunofluorescence. The data are presented as the mean ± SD (*n* = 10), the experiments were repeated three times, and statistical significance was determined by a *t* test. *P < 0.05, **P < 0.01 vs. normal; ^#^*P* < 0.05, ^##^*P* < 0.01 vs. the model.

To validate the mechanism of hesperetin preventing COPD and its progression to lung cancer, we investigated the expression of predicted core targets in lung tissue. Western blot analyses revealed that the protein expression of AKT1, IL6, VEGFA, MMP9 in lung tissue of COPD and lung cancer was significantly increased in the model group and hesperetin could dose-dependently prevent these protein expressions (*P* < 0.01) (Fig. 7a–b). However, hesperetin increased the expression of TP53 compared with the model group both in COPD and lung cancer (*P* < 0.01) (Fig. 7a–b). Additionally, immunofluorescence further confirms these results (Fig. 8), indicating a regulatory role of hesperetin in the protein expression of AKT1, IL6, VEGFA, MMP9 and TP53 in COPD and its progression to lung cancer.

## Discussion

*Chenpi* is a medicinal and food homology Chinese medicine and clinically used to treat cough associated with increased phlegm (25). As a drug commonly used in respiratory diseases, it has great development potential. In this study, we obtained five active ingredients from *chenpi* by network pharmacology, among which three compounds (hesperetin, naringenin,nobiletin) were potential materials for *chenpi* preventing COPD and its progression to lung cancer. In the previous report, naringenin, as an immunomodulator, could inhibit inflammatory response in diverse cell types (26); nobiletin could reduce the secretion of proinflammatory cytokines (27); while hesperetin has been shown to be an effective anti-cancer agent, such as promoting cell apoptosis and mitochondrial membrane potential loss in H522 cells, suppressing lung adenocarcinoma cell proliferation and migration (28–30). To further verify the key compound of *chenpi* preventing COPD and its progression to lung cancer, we carried out molecular docking between three compounds and the core targets. Although all three compounds had a good binding activity to these core targets, hesperetin showed the strongest binding activity with AKT1, IL6, VEGFA, MMP9 and TP53. Therefore, we speculate that hesperetin may be the most important active component of *chenpi* preventing COPD and its progression to lung cancer.

The PPI network indicated that AKT1, IL6, VEGFA, MMP9 and TP53 may be the core targets of *chenpi* preventing COPD and its progression to lung cancer. IL6 is an important pro-inflammatory cytokine (31, 32). Studies have shown that IL-6 at high concentrations could suppress the immune response and damage vascular endothelial cells (33, 34). VEGFA is a regulatory factor for angiogenesis and could directly promote the angiogenesis in tumour tissues and indirectly promote lymph node metastases (35). In addition, VEGF was found to be upregulated for inflammatory angiogenesis in lung tissues in COPD (36). MMP-9 plays important roles in degrading extracellular matrix and loss of epithelial cell integrity, and is closely related to tumour invasion and metastasis (37). AKT1, as an important downstream factor of the PI3K-AKT signal transduction, plays a key role in many physiological processes, such as cell proliferation, differentiation and apoptosis (38, 39). TP53 is one of the most well-known tumour suppressor and exerts multi-functional roles in controlling cell cycle checkpoints, apoptosis and DNA repair (40). In smokers with COPD, down-regulation of TP53 and p53-related signaling transduction may lead to lung tumorigenesis (41). Thus, these genes are reasonable as core targets for *chenpi* preventing COPD and its progression to lung cancer. In this study, we find up-regulation of AKT1, IL6, VEGFA, MMP9 but down-regulation of Tp53 in COPD and lung cancer affected mice, which confirmed the association of these core targets with COPD and its progression to lung cancer.

GO enrichment analysis showed that *chenpi* may play a role in anti-inflammation, antioxidant, immune regulation, cell apoptosis and proliferation by regulating oxidative reduction process, signal transfer, negative regulation of apoptotic process and cell proliferation. KEGG enrichment analysis showed that the core targets of the PPI network were mainly involved in the PI3K-Akt signaling pathway and MAPK signaling pathway. MAPK cascade activation is the centre of multiple signaling pathways (42, 43). Pharmacological study has showed that CSE-induced HIF-1 and MMP9 expression likely by activating MAPK signaling pathway (44). The PI3K-Akt signaling pathway can promote cell survival and growth in several ways, including regulation of apoptosis, proliferation and migration. Studies have found that an over activated PI3K-Akt signaling pathway can induce the activation of NF-кB and up-regulate the expression of pro-inflammatory cytokines IL-6 and TNF-α, resulting in persistent airway inflammation and accelerating the progress of COPD (45). Therefore, the PI3K-Akt signaling pathway and MAPK signaling pathway are reasonable as the core signaling pathway for *chenpi* preventing COPD and its progression to lung cancer.

Although network pharmacology provides a rough direction, substantial experimental data are necessary for verification. Thus, we validated the effects of hesperetin (the most important active component of *chenpi*) on COPD and its progression to lung cancer. In this study, we established a novel lung cancer using the COPD mouse model. At 4 weeks, COPD were formed successfully in the model group; at 8 weeks, lung cancer nodes were visible to the naked eye in the model group. Hesperetin treatment dose-dependently improved not only lung tissue injury in COPD but also carcinoma lesions in lung cancer. As expected, hesperetin could suppress the protein expression of AKT1, IL6, VEGFA, MMP9 and up-regulate the expression of Tp53, and thus reduce the risk of COPD and its progression to lung cancer.

## Conclusion

**Fig. 9:**
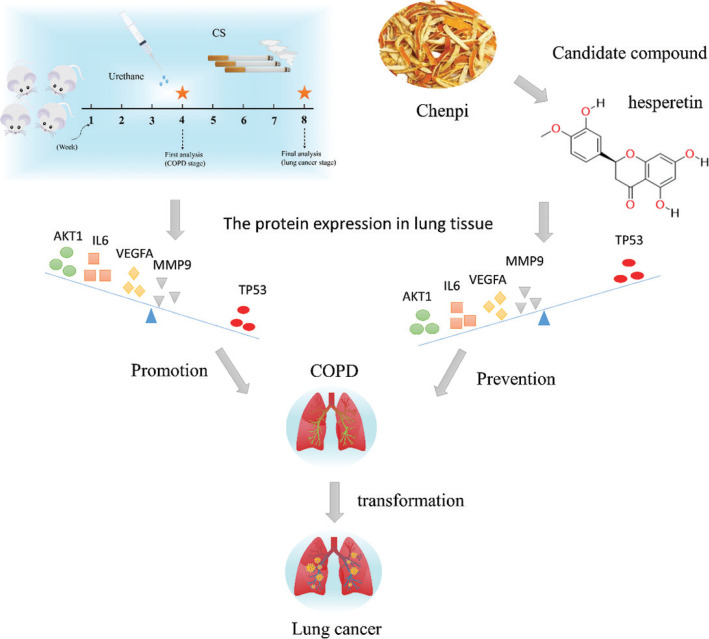
Hesperetin as a candidate compound of *chenpi* prevents COPD and its progression to lung cancer by regulating AKT1, IL6, VEGFA, MMP9 and TP53 in mouse lung cancer with COPD induced by CS and urethane.

In summary, our data suggested that hesperetin is a candidate compound of *chenpi* preventing COPD and its progression to lung cancer by regulating AKT1, IL6, VEGFA, MMP9 and TP53 (Fig. 9). At the same time, we also provided valuable clinical information for the long-term consumption of *chenpi* diets to decrease the risk of COPD and lung cancer. The limitation of *chenpi* is that contains excess of moisture and polysaccharides which are easily affected with mold in the process of storage. However, it has a good potential to help extract the key active components of *chenpi* for preventing COPD and its progression to lung cancer.
